# Cerebral blood flow assessment of preterm infants during respiratory therapy with the expiratory flow increase technique

**DOI:** 10.1016/j.rppede.2016.02.007

**Published:** 2016

**Authors:** Mariana Almada Bassani, Jamil Pedro Siqueira Caldas, Abimael Aranha Netto, Sérgio Tadeu Martins Marba

**Affiliations:** a Hospital da Mulher Professor Doutor José Aristodemo Pinotti, Centro de Atenção Integral à Saúde da Mulher (Caism), Universidade Estadual de Campinas (Unicamp), Campinas, SP, Brazil; b Departamento de Pediatria, Faculdade de Ciências Médicas, Universidade Estadual de Campinas (Unicamp), Campinas, SP, Brazil

**Keywords:** Newborn, Preterm, Physical therapy modalities, Blood flow velocity, Transcranial Doppler ultrasonography

## Abstract

**Objective:**

To assess the impact of respiratory therapy with the expiratory flow increase technique on cerebral hemodynamics of premature newborns.

**Methods:**

This is an intervention study, which included 40 preterm infants (≤34 weeks) aged 8-15 days of life, clinically stable in ambient air or oxygen catheter use. Children with heart defects, diagnosis of brain lesion and/or those using vasoactive drugs were excluded. Ultrasonographic assessments with transcranial Doppler flowmetry were performed before, during and after the increase in expiratory flow session, which lasted 5min. Cerebral blood flow velocity and resistance and pulsatility indices in the pericallosal artery were assessed.

**Results:**

Respiratory physical therapy did not significantly alter flow velocity at the systolic peak (*p*=0.50), the end diastolic flow velocity (*p*=0.17), the mean flow velocity (*p*=0.07), the resistance index (*p*=0.41) and the pulsatility index (*p*=0.67) over time.

**Conclusions:**

The expiratory flow increase technique did not affect cerebral blood flow in clinically-stable preterm infants.

## Resumo

**Objetivo:**

Avaliar a repercussão da fisioterapia respiratória com a técnica de aumento do fluxo expiratório sobre a hemodinâmica cerebral de recém-nascidos prematuros.

**Métodos:**

Estudo de intervenção no qual foram incluídos 40 neonatos prematuros (≤34 semanas) com 8-15 dias de vida, clinicamente estáveis em ar ambiente ou em uso de cateter de oxigênio. Foram excluídas crianças com malformações cardíacas, diagnóstico de lesão cerebral e/ou em uso de drogas vasoativas. Exames de ultrassonografia com avaliação por dopplerfluxometria cerebral foram feitos antes, durante e depois da sessão de aumento do fluxo expiratório, que durou cinco minutos. Foram avaliadas as velocidades de fluxo sanguíneo cerebral e os índices de resistência e pulsatilidade na artéria pericalosa.

**Resultados:**

A fisioterapia respiratória não alterou significativamente a velocidade de fluxo no pico sistólico (*p*=0,50), a velocidade de fluxo diastólico final (*p*=0,17), a velocidade média de fluxo (*p*=0,07), o índice de resistência (*p*=0,41) e o índice de pulsatilidade (*p*=0,67) ao longo do tempo.

**Conclusões:**

A manobra de aumento do fluxo expiratório não afetou o fluxo sanguíneo cerebral em recém-nascidos prematuros clinicamente estáveis.

## Introduction

The control of cerebral blood flow (CBF) involves complex neural and metabolic mechanisms, which are still immature in preterm newborns (PTNB).^1^ Therefore, these children show a failure in the autoregulation of the CBF, which is directly dependent on blood pressure and has a pattern known as pressure passive. The immaturity of cerebral blood flow control occurs mainly in newborns with gestational age up to 34 weeks, when the germinal matrix begins to involute.^2^ The subependymal matrix, which is located in the area adjacent to the lateral ventricles, is the site of proliferation of neuronal and glial cells,^2^^,^ 3 and is highly vascularized by irregular vessels with few structural support on its walls.^2^^,^ 4

Due to clinical reasons, newborns in intensive care are more likely to have CBF fluctuations, which increases the risk of hemorrhagic and ischemic cerebrovascular lesions, such as peri-intraventricular hemorrhage (PIVH) and periventricular leukomalacia (PVL), respectively. These neurological diseases may cause permanent motor sequelae of varying degrees, depending on the lesion extent, as well as cognitive, behavioral and intellectual disorders.^1^^,^ 3 ^-^ 5

CBF alterations are commonly associated with upper airway obstruction, severe respiratory diseases, apnea, hypoxia, hypercapnia, hypocapnia, ventilation with intermittent positive pressure, asynchrony with the ventilator, tracheal aspiration and expansion of circulating volume, as well as care routine, such as diaper changing and repositioning of the endotracheal tube, excessive manipulation and agitated sleep.^6^^-^ 8 As observed, the vast majority of situations that are known to cause significant alterations in CBF in preterm newborns is related to respiratory disorders resulting from pulmonary immaturity, leading to the need for increased hospital stay and ventilatory support and, consequently, increased risk of complications associated with mechanical ventilation and increased morbidity and mortality.

In this context, respiratory physiotherapy has become necessary and a routine in most neonatal intensive care units (NICU) worldwide.^9^ The main objectives of respiratory physiotherapy are the prevention and treatment of bronchial obstruction due to accumulation of secretion, which contributes to reducing its deleterious effects, such as hyperinflation, atelectasis, changes in the ventilation-perfusion and increased respiratory effort.^10^^,^ 11

Few studies have investigated the influence of respiratory physiotherapy on brain injuries in preterm newborns regarding CBF alterations.^12^^-^ 15 To date, no study quantitatively described the pattern of brain hemodynamic behavior in this population before, during and after respiratory physiotherapy maneuvers are performed.

The objective of this study was to assess the influence of physiotherapy on the CBF in clinically-stable preterm newborns.

## Method

This is an intervention study, carried out in intensive care and neonatal intermediate care units at Hospital da Mulher Prof. Dr. José Aristodemo Pinotti, Centro de Atenção Integral à Saúde da Mulher (Caism) of Universidade Estadual de Campinas (Unicamp), from October 2013 to June 2014. The sample consisted of clinically-stable preterm newborns with gestational age ≤34 weeks, between 8 and 15 days of postnatal age, spontaneously breathing room air or receiving oxygen with the aid of a nasal cannula and with no contraindications for respiratory physiotherapy and the supine position. In our service, all preterm newborns, even without pulmonary involvement, have indication for respiratory physiotherapy; however, in the ones that require oxygen and/or who have pulmonary and/or nasal secretions, respiratory physiotherapy is intensified. We excluded all newborns with cardiac and/or neurological malformations, diagnosis of brain lesion (hemorrhagic or ischemic) during any period of hospitalization and/or receiving vasoactive drugs. Written informed consent was obtained from the parents/tutors of each subject.

To calculate the sample size, a pilot study was performed, in which 10 preterm newborns underwent identical assessments to those used for the data collection in this study (described later). Considering a type I error of 5% and a type II error of 20%, the sample size was determined by the difference between the means of paired measurements for all studied variables. Thus, a sample size of 40 newborns was determined.

Gestational age (GA) was obtained from the date of the last menstrual period. If this date was unknown or uncertain, the GA estimated at an early ultrasound (less than or equal to 16 weeks) and/or clinical and neurological examination of the newborn by New Ballard method was considered.^16^ We did not consider the corrected age.

CBF assessment of was carried out by transfontanellar Doppler ultrasonography, performed by the same neonatologist, in order to avoid observer-related variations. The Sono Site™ system, M-Turbo model, with a 5MHz transducer was used. The pericallosal artery, a branch of the anterior cerebral artery, adjacent to the knee of the corpus callosum was evaluated. This artery was chosen because it is widely used in scientific studies for similar tests,^17^ as well as its easy access through the anterior fontanelle. The following were assessed: mean flow velocity (MFV), peak systolic flow velocity (PSV), end-diastolic flow velocity (EDFV), index of resistance (IR=PSV−EDFV/PSV) and pulsatility index (PI=PSV−EDFV/MFV).

The examinations were carried out with the newborn in the supine position with the head in midline position, and kept at rest or reduced motor activity for approximately 10min, after which the brain ultrasound was performed to exclude brain lesions and, subsequently, the first Doppler ultrasound examination (*T*_0_) was performed. After that, the expiratory flow increase (EFI) maneuvers were performed for 5min. The examination was repeated on the second (*T*_1_) and fifth (*T*_2_) minutes of respiratory physiotherapy and 10min after completion of the maneuvers (*T*_3_). After the tests, the newborns received nursing care, according to the service routine. The examinations were performed one hour before the following feeding to prevent vomiting and bonchoaspiration. Fasting patients and those fed by continuous infusion through infusion pump or parenteral nutrition were assessed one hour before the subsequent nursing care in order to not interrupt the newborn's resting and sleep time. Newborns that were restless or tearful during the physiotherapy session were calmed with a 25% sucrose solution and/or non-nutritive sucking with pacifier or a gloved finger. All routine brain ultrasonographic examinations, performed after the tests carried out for this study, were systematically followed by the researchers.

**Figure 1:**
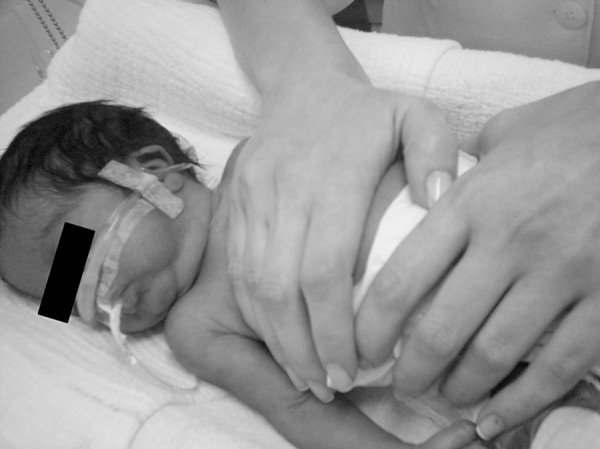
Respiratory physiotherapy technique of expiratory flow increase in preterm newborns.

The chosen physiotherapy technique was the slow EFI method adapted to premature patients, which was applied by the same therapist in a consistent and standardized manner. This maneuver consists in slowly applying a slight pressure on the patient's chest with one hand, obliquely (cephalocaudal and anteroposterior planes), starting at the end of the inspiratory plateau and ending at the end of expiration, which is prolonged. The hand must be positioned between the sternal notch and the xiphoid process of the newborn's sternum. The other hand of the therapist is placed on the last ribs (without applying pressure) as a bridge, of which columns are the thumb and forefinger (or middle finger) (Fig. 1). Thus, contact with the child's abdomen is avoided and the expansion of the lower ribs is limited, allowing better diaphragm excursion and preventing an increase in intraabdominal pressure.^2^^,^ 18 ^,^ 19 In this study, this procedure was repeated for 5min, with a brief pause in the middle of the period to perform the examination. This respiratory physiotherapy technique was chosen because it is currently one of the most often used in our service.

Maternal and neonatal sociodemographic variables were collected from medical records to characterize the sample. The absolute frequency and percentage of qualitative variables and descriptive statistics of quantitative variables, with measures of central tendency, position and dispersion (mean, standard deviation and minimum and maximum values) were computed.

Heart rate and oxygen saturation were continuously monitored in a multi-parameter monitor. Respiratory frequency was assessed by visual count. Blood pressure (BP) was measured non-invasively using the automated oscillometric method with a multi-parameter monitor, preferably in the right upper limb.

To investigate whether respiratory physiotherapy promoted alterations in CBF, analysis of variance for repeated measures was used, with the response variables transformed into ranks. Each child served as his/her own control. The twinning variable was used to adjust the models in order to avoid statistical bias, since newborn twins do not have independent obstetric history. The statistical significance level was set at *p*<0.05. The statistical analysis was carried out using The SAS System for Windows, version 9.2 (2002-2008).

The Protocol for this study was approved by our hospital's Institutional Research Committee and by the University's local Ehtics Committee, under protocols no. 24/2013 and 421.237, respectively.

## Results

Forty-two newborns were enrolled in this study, of which two were excluded due to the presence of periventricular leukomalacia (PVL), observed in pre-discharge transfontanellar ultrasound. Thus, 40 newborns were included, of which most of them (95%) were breathing room air, were males (52.5%) and born via Cesarean section (67.5%). The mean gestational and postnatal age was 31.8±1.6 weeks (range: 28-34) and 10.9±1.9 days (range: 8-15), respectively. The newborns had a mean birth weight of 1658±539g, ranging from 830 to 3840g. Fifteen newborns (37.5%) had birth weight<1500g. Most patients (70%, *n*=28) were considered adequate for gestational age, while 27.5% (*n*=11) were small for gestational age and only one (2.5%) was considered large for gestational age. On examination, the patients weighed on average 1617±519g, ranging from 840 to 3900g. Of the newborns, 10 (25%) were twins. Regarding the maternal history, 12 (30%), four (10%) and seven (17.5) were born to hypertensive, smoker and diabetic mothers, respectively. Eight (20%) patients were born to mothers that had used magnesium sulfate and 33 (82.5%) to mothers who received antenatal corticosteroids. The most frequently observed neonatal morbidities were risk of ovular infection (*n*=20, 50%), respiratory distress (*n*=36, 90%), use of surfactant (*n*=20, 50) and patent ductus arteriosus (*n*=9, 22.5%).

**Figure 2:**
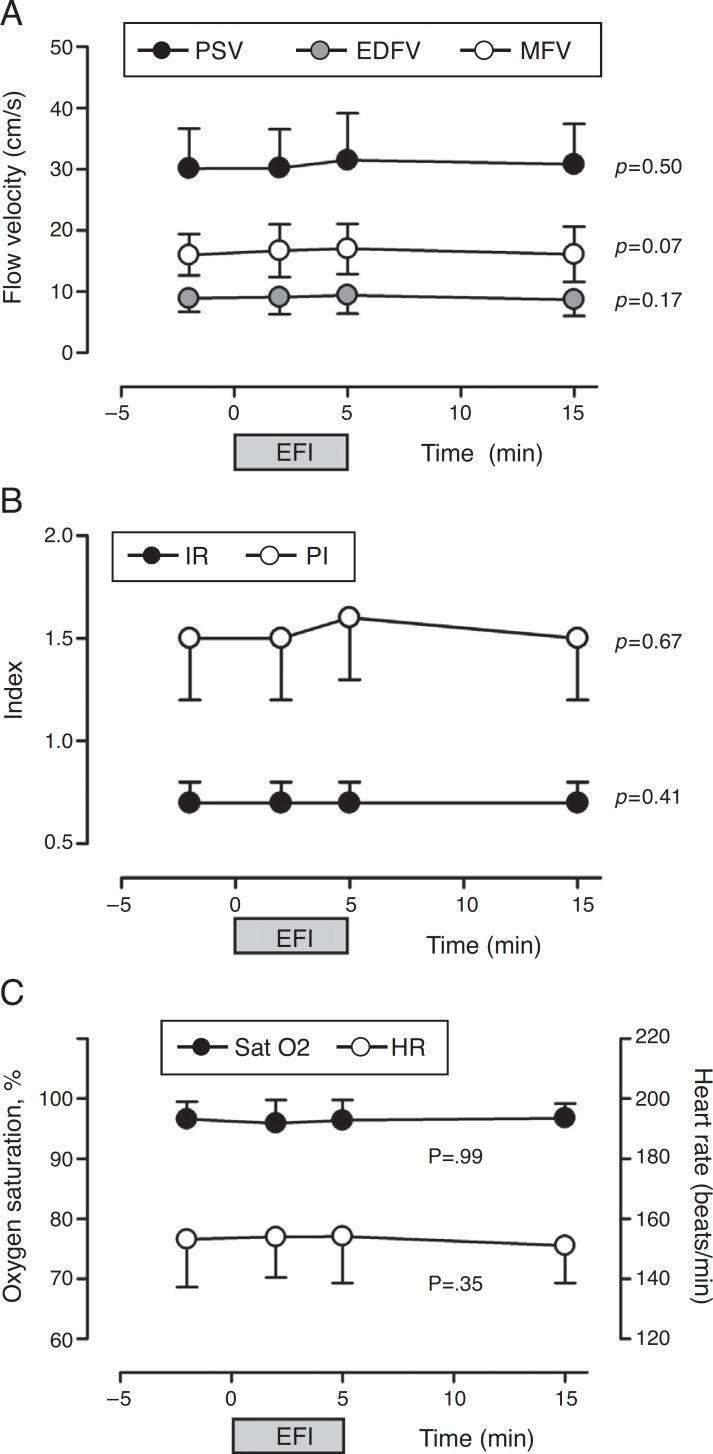
Influence of physiotherapy on cerebral blood flow velocity (A), resistance and pulsatility indexes (B) and oxygen saturation and heart rate (C) of premature newborns (*n*=40). PSV, peak systolic flow velocity; MFV, mean flow velocity; EDFV, end-diastolic flow velocity; PI, pulsatility index; IR, index of resistance; SatO_2_, peripheral oxygen saturation; HR, heart rate; EFI, expiratory flow increase technique.

No assessed CBF variable (PSV, EDFV, MFV, IR and PI) was significantly altered by the respiratory physiotherapy maneuvers over time (*T*_0_, *T*_1_, *T*_2_ and *T*_3_) (Fig. 2).

Hemoglobin oxygen saturation (*p*=0.99) and heart rate (*p*=0.07) did not change significantly with the respiratory physiotherapy over time and were within normal limits (Fig. 2). Systolic (SBP, 70.5±17.5), diastolic (DBP, 39.7±11.8) and mean blood pressure levels (MBP, 47.7±10.3), as well as respiratory rate (RR, 49.2±10.5) were also within the normal limits^20^ and, after the physiotherapy session, were similar to previous values at *T*_0_ (RR: 53.5±10.5rpm; SBP: 68.8±12.3mmHg; DBP: 38.2±10.3mmHg; MBP: 46.1±7.5mmHg).

## Discussion

This study showed, in an unprecedented manner, that respiratory physiotherapy using the EFI technique did not significantly change the CBF velocities, and did not seem to modify cerebral vascular resistance in clinically-stable preterm newborns. Some studies investigated the association between respiratory therapy and the occurrence of cerebrovascular lesions.^12^^-^ 15 However, to date, we are unaware of the existence of studies that evaluated CBF velocity parameters in preterm newborns before, during and after some type of respiratory physiotherapy maneuver.

In the late 1990s, Harding et al.^15^ observed a significant occurrence of encephaloclastic porencephaly in extremely-low birth weight newborns and identified respiratory physiotherapy as one of the predisposing factors. At the time, these results generated great controversy and, after that, further studies on this subject were performed, which showed no association between respiratory physiotherapy maneuvers and brain damage in preterm newborns.^12^^-^ 14 ^,^ 21 However, these studies assessed different physiotherapy techniques, such as vibration and percussion associated or not to tracheal aspiration. Only one study addressed the association of EFI technique with the occurrence of PVL and PIVH in preterm newborns. The authors concluded that the use of EFI does not increase the incidence of brain lesions in newborns with respiratory failure, and that it does not worsen preexisting lesions.^12^

Cerqueira-Neto et al.^22^ studied the cerebral hemodynamics of 20 adult patients with severe head trauma during respiratory physiotherapy (chest vibration, EFI and endotracheal aspiration with saline instillation). The patients were sedated, received analgesics and neuromuscular blocker, were intubated and on invasive mechanical ventilation. The intracranial pressure (ICP) of those patients was monitored by an intraventricular catheter. The authors observed that physiotherapy maneuvers of thoracic vibration and EFI did not result in significant effects on ICP, mean airway pressure (MAP) and cerebral perfusion pressure. However, endotracheal aspiration significantly increased ICP and MAP, which returned to baseline levels within 10min. The authors suggest that the increase in ICP may be associated with increased MAP and with the limitation of cerebral blood flow autoregulation, which can occur in patients with severe head trauma,^23^ as well as in preterm newborns.^1^ Another relevant study is the one by Maynard et al.,^24^ who assessed the pattern of CBF velocity in the middle cerebral artery before, during and after a rapid thoracic compression (used for pulmonary function testing, but which can be comparable to the rapid EFI technique) in 12 preterm newborns and full-term newborns. The authors observed a significant increase in EDFV and PI reduction during the maneuver, with returned to baseline values immediately after thoracic release. Sneezing and soft vocalizations produced changes similar to those observed during rapid thoracic compression. In this study, some newborns had sneezing and/or coughing during the physiotherapy session, which is expected. A limitation of this study is the fact that we did not test the isolated influences of crying, coughing, sneezing, hiccups and vocalizations. In addition, due to technical difficulties, cerebral blood flow measurements were not taken at the exact moment of chest compression.

As currently the EFI technique is widely used in the NICU, we consider of great importance to quantitatively assess its effects on the CBF in premature newborns, as this maneuver involves compression, even slight and slow, of a more compliant chest. It is not known whether this situation could change the intrathoracic pressure enough to affect venous return and, thus, change CBF. We also emphasize that the technique used in premature newborns does not involve the use of abdominal pressure, which, in the original technical description, should be applied in the opposite direction to that of thoracic pressure.^18^ It is possible that the pressure on the abdomen may significantly increase intra-abdominal pressure and, consequently, the intrathoracic pressure and ICP, but this possibility has yet to be demonstrated. It is noteworthy that the physiotherapy team in our service only treats preterm newborns after 72h of life. There is great concern about causing CBF changes in preterm newborns, particularly those with lower GA and birth weight (<1.500g), due to the hemodynamic instability in the postnatal period, particularly within the first 72h,^25^^-^ 28 during which greater fluctuations of brain circulation occur due to pressure passive autoregulation,^27^ and hence increasing the risk of hemorrhagic (HPIV) and/or ischemic (PVL)^1^ cerebrovascular lesions and their complications. However, there is evidence that the CBF is still stabilized during the first and second weeks of life,^29^^,^ 30 which justifies the investigation of the respiratory physiotherapy influence in children with postnatal age of 8-15 days. A limitation of this study is that it did not consider the corrected gestational age of patients at the time of the procedure. We chose to use chronological age as a risk factor for change in the outcome, as that was the criterion for subject selection. Nevertheless, only four patients had corrected age>34 weeks at the examination.

Not all patients in this study had an absolute indication for respiratory physiotherapy, as the majority was in a clinically-stable situation and with no oxygen support. On the other hand, the therapy was indicated for oxygen-dependent patients and for those with pulmonary and/or nasal secretion. It is worth mentioning that no subject had contraindications for such intervention, which would not bring any harm to the patient. Therefore, the study population was heterogeneous regarding the indication of pulmonary therapy, but stable considering the clinical point of view.

Furthermore, it is important to mention that the children were not followed after hospital discharge. Some patients were discharged right after the assessment for this study. Early discharges were due to favorable clinical outcome or inter-hospital transfer, which was the case for 18 of the 40 studied children. Therefore, we have no information about the possible development of subsequent brain lesions.

The knowledge of factors that could change the CBF of preterm newborns is important for the prevention and control of complications related to prematurity, such as cerebrovascular lesions. As the vast majority of premature newborns need respiratory physiotherapy, it is important to know the pattern of cerebral hemodynamics in these patients during the application of the most often used physiotherapy techniques. This information can contribute to better care for the newborns, as well as better prevention, treatment and control of cerebral disorders and complications that affect them. Additionally, it may help physiotherapists for a more adequate and safer indication of respiratory therapy.

It can be said that the EFI is a safe physiotherapy technique when applied in clinically-stable preterm newborns, as it did not result in significant changes in CBF. In this study, we analyzed clinically-stable newborns as a first step toward the assessment of the technique safety. The extrapolation of these results in clinically-unstable infants and on the first days of life should be viewed with caution and becomes an interesting field for further studies addressing critically-ill preterm newborns.
